# Low vitamin D status is associated with more depressive symptoms in Dutch older adults

**DOI:** 10.1007/s00394-015-0970-6

**Published:** 2015-07-04

**Authors:** E. M. Brouwer-Brolsma, R. A. M. Dhonukshe-Rutten, J. P. van Wijngaarden, N. L. van der Zwaluw, E. Sohl, P. H. In’t Veld, S. C. van Dijk, K. M. A. Swart, A. W. Enneman, A. C. Ham, N. M. van Schoor, N. van der Velde, A. G. Uitterlinden, P. Lips, E. J. M. Feskens, L. C. P. G. M. de Groot

**Affiliations:** Division of Human Nutrition, Wageningen University, P.O. Box 8129, 6700 EV Wageningen, The Netherlands; Department of Epidemiology and Biostatistics and the EMGO Institute for Health and Care Research, VU University Medical Centre, Van der Boechorststraat 7, 1081 BT Amsterdam, The Netherlands; Department of Internal Medicine, Erasmus University Medical Centre, P.O. Box 2040, 3000 CA Rotterdam, The Netherlands; Department of Internal Medicine, Section of Geriatrics, Academic Medical Center, University of Amsterdam, Meibergdreef 9, 1105 AZ Amsterdam, The Netherlands; Department of Internal Medicine, Endocrine Section, VU University Medical Center, P.O. Box 7057, 1007 MB Amsterdam, The Netherlands

**Keywords:** Vitamin D, Vitamin D receptor polymorphisms, Depression, Elderly, Diabetes

## Abstract

**Purpose:**

The existence of vitamin D receptors in the brain points to a possible role of vitamin D in brain function. We examined the association of vitamin D status and vitamin D-related genetic make-up with depressive symptoms amongst 2839 Dutch older adults aged ≥65 years.

**Methods:**

25-Hydroxyvitamin D (25(OH)D) was measured, and five ‘vitamin D-related genes’ were selected. Depressive symptoms were measured with the 15-point Geriatric Depression Scale. Results were expressed as the relative risk of the score of depressive symptoms by quartiles of 25(OH)D concentration or number of affected alleles, using the lowest quartile or minor allele group as reference.

**Results:**

A clear cross-sectional and prospective association between serum 25(OH)D and depressive symptom score was observed. Fully adjusted models indicated a 22 % (RR 0.78, 95 % CI 0.68–0.89), 21 % (RR 0.79, 95 % CI 0.68–0.90), and 18 % (RR 0.82, 95 % CI 0.71–0.95) lower score of depressive symptoms in people in the second, third, and fourth 25(OH)D quartiles, when compared to people in the first quartile (*P* for trend <0.0001). After 2 years of daily 15 µg vitamin D supplementation, similar associations were observed. 25(OH)D concentrations did not significantly interact with the selected genes.

**Conclusion:**

Low serum 25(OH)D was associated with higher depressive symptom scores. No interactions between 25(OH)D concentrations and vitamin D genetic make-up were observed. In view of the probability of reverse causation, we propose that the association should be further examined in prospective studies as well as in randomized controlled trials.

**Electronic supplementary material:**

The online version of this article (doi:10.1007/s00394-015-0970-6) contains supplementary material, which is available to authorized users.

## Introduction

Globally almost 350 million people are affected by depression [1]. Depression is regularly accompanied by a reduced quality of life, a variety of comorbidities, and a higher mortality rate [2]. The high prevalence of depression [1], its possible consequences [2], plus the unwanted side effects [3] that often accompany the use of anti-depressive medication, indicate the need for preventive measures.

One of the factors that have been suggested to beneficially influence mood and depression is sunlight [4]. Sunlight, specifically ultraviolet-B radiation, may positively reduce depressive symptoms by activating the vitamin D synthesis in the skin. Mechanistic studies on brain function support this hypothetical pathway (reviewed in [5, 6]). Furthermore, as low vitamin D concentrations have been associated with diabetes [7], and diabetes with depression [8], vitamin D may also indirectly affect the prevalence of depressive symptoms by influencing glucose tolerance. In addition, it has been postulated that vitamin D deficiency makes the brain more susceptible for neurobiological triggers, like diabetes. Thus, both interaction and modification effects by diabetes may be observed when examining the association between vitamin D and depression.

One of the groups at risk for a vitamin D deficiency is the elderly population, which may be explained by their reduced skin capacity to synthesize vitamin D, reduced outdoor activities, and decreased dietary intake. Of seven observational studies investigating the potential association between 25(OH)D and depression in populations aged ≥60 years [9–15], five studies observed significant associations [11–15], indicating that persons with higher 25(OH)D concentrations had a lower probability of being depressed when compared to those with lower 25(OH)D concentrations. Even though most of these studies are in favour of vitamin D, it needs to be emphasized that there is considerable heterogeneity between studies due to differences in study design, populations, sample sizes, covariates adjusted for, and method to quantify depression. Moreover, specific pathways explaining the association between 25(OH)D and depression in these populations have not been investigated. Thus, more—and more detailed—evidence is warranted.

More detailed evidence may arise from studies that take into account genetic variation in vitamin D-related genes. A large genome-wide association study namely observed significant differences in 25(OH)D status according to variation in genes that have been linked with vitamin D synthesis (i.e. DHCR7 and CYP2R1) and vitamin D metabolism (i.e. CYP24A1 and GC) [16]. Next to these genes that may influence 25(OH)D status, associations between 25(OH)D and depression may also be modified by the efficiency of the vitamin D receptor (VDR), which has been identified in brain tissue [17]. Potentially interesting polymorphisms of the VDR gene include TaqI/BsmI, ApaI, and Cdx2 [18].

In the current study, we investigated the cross-sectional association between serum 25(OH)D and the score of depressive symptoms in a large sample of older adults. To further elucidate the effect of temporality, we also explored the association between baseline 25(OH)D concentrations and the score of depressive symptoms after 2 years of daily 15 µg vitamin D_3_ supplementation. In addition, interactions between 25(OH)D and vitamin D-related genes were examined, specifically DHCR7, CYP2R1, CYP24A1, GC, TaqI/BsmI, ApaI, and Cdx2. Finally, to further investigate the potential underlying mechanisms, also the potential modification and mediation effects of self-reported diabetes were studied.

## Methods

### Participants

**Fig. 1:**
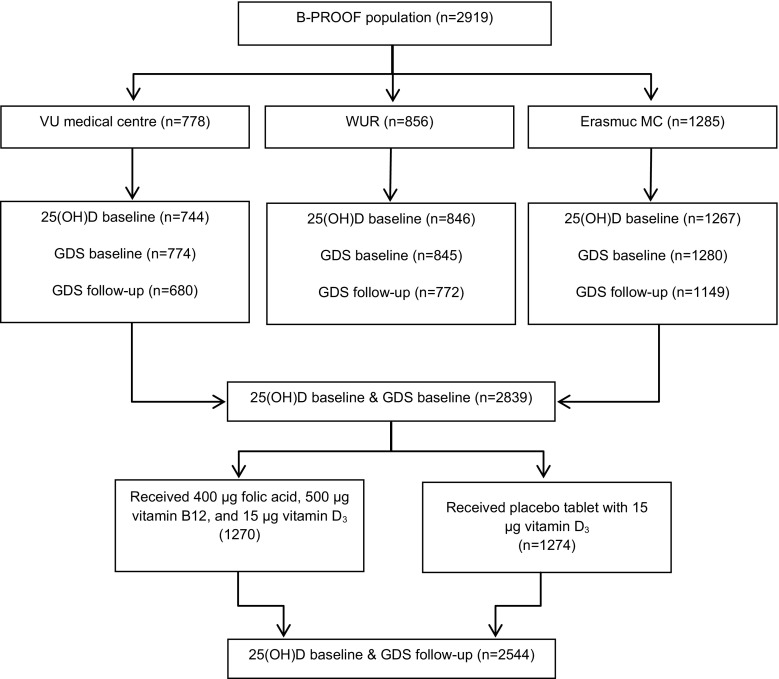
Flowchart of B-PROOF study data used for the cross-sectional and prospective analyses on 25(OH)D concentrations and depression scores. GDS indicates geriatric depression score-15 questionnaire

This study was performed using data of the B-PROOF study; a randomized, double-blind, placebo-controlled trial designed to assess the efficacy of 2-year daily oral supplementation of vitamin B_12_ and folic acid on fractures in mildly hyperhomocysteinemic (plasma homocysteine 12–50 µmol/l) community-dwelling older adults aged ≥65 years. Given the known beneficial effect of vitamin D on bone health, 15 µg vitamin D_3_ was added to both placebo and treatment tablets. Participants were mainly recruited via registries of municipalities in the area of the research centres; all inhabitants aged ≥65 years were invited by regular mail. In addition, participants were recruited by means of information brochures, and meetings that were organized for elderly home residents in the area of Rotterdam, Amsterdam, and Wageningen. Finally, also potential eligible adults who participated in previous studies of the research centres were contacted. For the current analyses, only data on baseline 25(OH)D status were available; therefore, the impact of the supplementation regimen on 25(OH)D status over 2 years could not be verified by checking the impact on 25(OH)D concentrations. However, based on the analyses on the primary outcome of the B-PROOF study, we do know that 2661 participants in this study complied with taking ≥80 % of the study tables, including 91.4 % of participants in the intervention group and 90.9 % in the placebo group [19]. At baseline, 25(OH)D status and depression data were available of 2839 participants. After 2 years of follow-up, depression data were available for 2544 participants. Figure 1 shows a detailed overview of the participant flow. Details on the study design of this trial have been reported previously [20]. The Medical Ethics Committee of Wageningen UR approved the study protocol, and the Medical Ethics Committees of VUmc and Erasmus MC confirmed local feasibility. All participants gave written informed consent.

### Biochemical analyses and genotyping

Blood samples were drawn in the morning when participants were fasted or had consumed a restricted breakfast. Samples were stored at −80 °C until determination. Serum 25(OH)D was measured by isotope dilution–online solid-phase extraction–liquid chromatography–tandem mass spectrometry (ID-XLC-MS/MS) [21]. DNA was isolated from buffy coats. Samples were genotyped for about 700,000 SNPs using the Illumina Omni Express array, covering >90 % of all common variation in the genome. Genes selected for this study included genes affecting vitamin D synthesis [i.e. DHCR7 (rs12785878) and CYP2R1 (rs10741657)], vitamin D metabolism [CYP24A1 (rs6013897) and GC (rs2282679)], and vitamin D receptor (VDR) action (VDR rs731236 [TaqI], VDR rs1544410 [BsmI], VDR rs7975232 [ApaI], and VDR rs11568820 [Cdx2]).

### Mental health

The 15-item Geriatric Depression Scale (GDS) is a widely used self-report questionnaire, which is designed to measure the number of depressive symptoms in an elderly population [22]. Scores can range from 0 up to 15 points, where higher scores are indicative of more depressive symptoms. Scores ranging from 5 to 7 are suggestive of mild depression, scores ranging from 8 to 9 are indicative of moderate depression, and scores ≥10 suggest severe depression [22].

### Covariates

Height was measured at baseline with a stadiometer to the nearest 0.1 cm. Weight was measured to the nearest 0.5 kg with a calibrated analogue scale. Body mass index (BMI) was calculated as weight/height^2^. Data on education level (primary, secondary, higher), smoking status (no, current, former), physical activity (kcal/day) [23], alcohol consumption (light, moderate, excessive) [24], and disease history (i.e. diabetes, hypertension, cardiac disease, stroke/TIA) were collected by means of questionnaires. Season was based on the month of blood sampling. Season of blood collection was dichotomized in summer/fall (June–November) and winter/spring (December–May).

### Statistical analyses

Participants characteristics are reported as mean with standard deviation (SD), or as percentages. Medians with interquartile range were used to report skewed variables. Restricted cubic spline regression was used to visualize the dose–response between serum 25(OH)D and the depressive symptom score, where the model was adjusted for age, sex, BMI, education, smoking, physical activity, alcohol intake, season of blood sampling, centre, and self-reported diabetes. The association between serum 25(OH)D and depression symptom score was further explored using multiple Poisson’s regression, providing relative risks (RRs). These RRs correspond to the probability of reporting a higher score of depressive symptoms when allocated to the second, third, or fourth quartile of this population compared with participants allocated to the first quartile (i.e. those with the lowest 25(OH)D concentrations). Analyses were adjusted for age, sex (model 1), BMI, education, smoking, alcohol intake, physical activity, season of blood sampling, centre (model 2), and diabetes (model 3). Follow-up analyses with model 2 and model 3 were additionally adjusted for treatment.

Poisson’s regression was also used to test whether self-reported diabetes and vitamin D-related genes were associated with the score of depressive symptoms. Associations between self-reported diabetes and the score of depressive symptoms were adjusted for age, sex, BMI, education, smoking, physical activity, alcohol intake, and centre.

Cox proportional hazards regression was applied to examine associations between serum 25(OH)D and self-reported diabetes. By assigning a constant risk period to all participants in the study, the obtained hazard ratio can be considered as a prevalence ratio (PR) [25]. This PR corresponds to the probability of having diabetes when allocated to the second, third, or fourth quartile of serum 25(OH)D, compared with participants allocated to the first quartile of serum 25(OH)D (i.e. lowest serum 25(OH)D concentrations). These analyses were adjusted for age, sex (model 1), BMI, education, smoking, physical activity, alcohol intake, season of blood sampling, and centre (model 2).

Finally, Poisson’s regression was conducted to examine potential interactions between 25(OH)D and self-reported diabetes, and serum 25(OH)D and vitamin D-related genes. All analyses were performed using the statistical package SAS, version 9.1 (SAS Institute Inc., Cary, NC, USA), except for the restricted cubic spline regression, which was analysed in the program R.

## Results

Population characteristics are presented in Table 1. Participants were on average 74.1 ± 6.5 years old and had a mean serum 25(OH)D concentration of 56 ± 25 nmol/L; 45 % had a concentration below 50 nmol/L, 7 % (baseline)/8 % (follow-up) of the participants had ≥5 depressed symptoms, and 10 % reported to have diabetes. Over quartiles of serum 25(OH)D, significant differences were observed for age, sex, BMI, smoking habits, alcohol consumption, physical activity level, season of blood sampling, score of depressive symptoms, self-reported diabetes, DHCR7, and CYP2R1.

**Table 1:** Characteristics of Dutch older adults participating in the B-PROOF study per quartile of serum 25(OH)D (nmol/L)

	*Q*1	*Q*2	*Q*3	*Q*4	*P* value
	<36.7	36.7–53.4	53.4–71.7	71.7>	
*N* ^a^	711	718	712	716	
25(OH)D (nmol/L)	26 ± 7	45 ± 5	62 ± 5	89 ± 15	<0.0001
Sex, number of men (%)	306 (43)	365 (51)	383 (54)	374 (52)	0.0002
Age, years	76.1 ± 7.4	73.7 ± 6.3	73.7 ± 6.1	72.7 ± 5.7	<0.0001
Body mass index (kg/m^2^)	27.7 ± 4.6	27.3 ± 3.9	27.3 ± 3.7	26.3 ± 3.5	<0.0001
Smoking status [*n* (%)]					0.02
Non-smoker	266 (37)	240 (33)	232 (33)	231 (32)	
Smoker	83 (12)	72 (10)	66 (9)	56 (8)	
Former smoker	362 (51)	406 (57)	414 (58)	429 (60)	
Physical activity (kcal/day)	544 ± 385	681 ± 504	679 ± 539	693 ± 451	<0.0001
Educational level [*n* (%)]					0.07
Primary education	393 (55)	309 (54)	386 (54)	347 (48)	
Secondary education	142 (20)	151 (21)	155 (22)	152 (21)	
Higher education	176 (25)	177 (25)	171 (24)	217 (31)	
Alcohol intake					0.001
Light	512 (72)	509 (71)	446 (63)	456 (64)	
Moderate	168 (24)	188 (26)	239 (34)	228 (32)	
Excessive	29 (4)	21 (3)	27 (3)	32 (4)	
GDS baseline	1 (3)	1 (2)	1 (2)	1 (2)	<0.0001
(% GDS score ≥ 5)	(11)	(5)	(6)	(6)	
GDS follow-up	1 (3)	1 (2)	1 (2)	1 (2)	<0.0001
(% GDS score ≥5)	(11)	(5)	(9)	(7)	
Diabetes [*n* (%)]	79 (14)	55 (10)	54 (10)	43 (8)	0.01
Hypertension [*n* (%)]	233 (41)	232 (41)	203 (37)	195 (37)	0.25
TIA/stroke [*n* (%)]	74 (13)	33 (6)	42 (8)	44 (8)	0.0002
Cardiac disease [*n* (%)]	166 (29)	138 (25)	130 (24)	123 (23)	0.08
Blood sampling in summer [*n* (%)]	224 (32)	324 (45)	429 (60)	524 (73)	<0.0001
Assigned to B12 and folic acid supplementation [*n* (%)]	372 (52)	359 (50)	329 (46)	373 (52)	0.07
TaqI/BsmI (*n* = 2555)					0.75
0 affected alleles [*n* (%)]	108 (18)	113 (18)	110 (17)	127 (20)	
1 affected alleles [*n* (%)]	285 (48)	315 (49)	316 (49)	318 (49)	
2 affected alleles [*n* (%)]	206 (34)	211 (33)	173 (34)	198 (31)	
ApaI (*n* = 2555)					0.55
0 affected alleles [*n* (%)]	137 (23)	141 (22)	155 (24)	134 (21)	
1 affected alleles [*n* (%)]	299 (50)	328 (51)	302 (46)	316 (49)	
2 affected alleles [*n* (%)]	163 (27)	170 (27)	192 (30)	193 (30)	
Cdx2 (*n* = 2555)					0.99
0 affected alleles [*n* (%)]	21 (4)	23 (4)	25 (4)	25 (4)	
1 affected alleles [*n* (%)]	180 (30)	201 (31)	193 (30)	200 (31)	
2 affected alleles [*n* (%)]	398 (66)	415 (65)	431 (66)	418 (65)	
DHCR7 (*n* = 2555)					0.004
0 affected alleles [*n* (%)]	50 (8)	59 (9)	49 (7)	37 (6)	
1 affected alleles [*n* (%)]	261 (44)	252 (40)	232 (36)	233 (36)	
2 affected alleles [*n* (%)]	288 (48)	328 (51)	368 (57)	373 (58)	
CYP2R1 (*n* = 2555)					0.01
0 affected alleles [*n* (%)]	112 (19)	97 (15)	98 (15)	116 (18)	
1 affected alleles [*n* (%)]	251 (42)	307 (48)	337 (52)	318 (49)	
2 affected alleles [*n* (%)]	236 (39)	235 (37)	214 (33)	209 (33)	
CYP24A1 (*n* = 2555)					0.06
0 affected alleles [*n* (%)]	35 (6)	25 (4)	25 (4)	25 (4)	
1 affected alleles [*n* (%)]	218 (36)	198 (31)	226 (35)	199 (31)	
2 affected alleles [*n* (%)]	346 (58)	416 (65)	398 (61)	419 (65)	
GC (*n* = 2555)					<0.0001
0 affected alleles [*n* (%)]	75 (13)	62 (10)	55 (8)	20 (3)	
1 affected alleles [*n* (%)]	234 (39)	278 (43)	252 (39)	215 (33)	
2 affected alleles [*n* (%)]	290 (48)	299 (47)	342 (53)	408 (64)	

Values are expressed as a mean ± SD, median (IQR) or *n* (%). Chi-squared tests for categorical variables and one-way analysis of variance for continuous variables were performed to compare participant characteristics over quartiles of 25(OH)D

^a^Dropout after 2 years of follow-up: *Q*1 (*n* = 94), *Q*2 (*n* = 75), *Q*3 (*n* = 74), and *Q*4 (*n* = 52)

### Serum 25(OH)D, vitamin D-related genetic make-up, and score of depressive symptoms

**Fig. 2:**
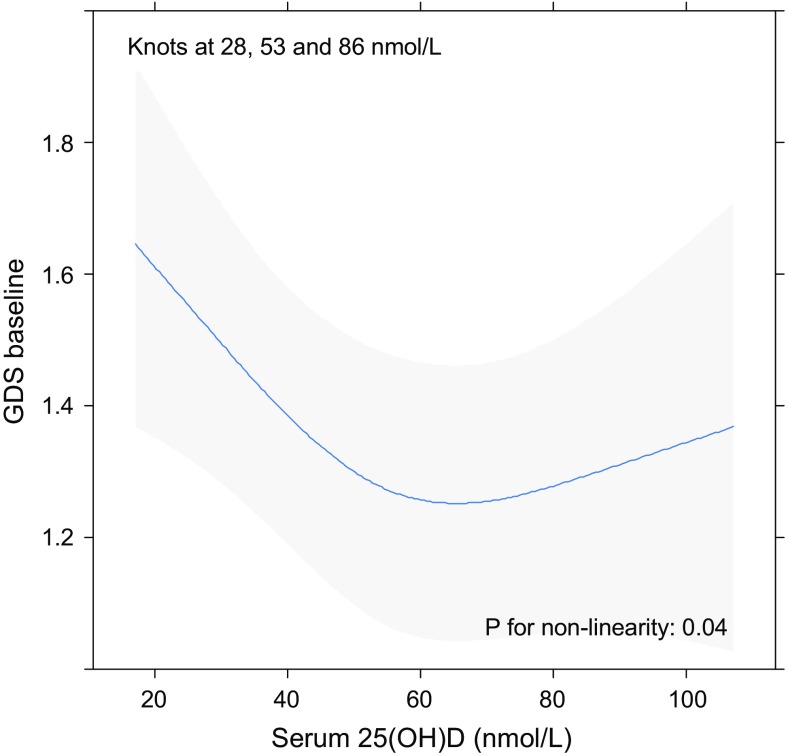
Cross-sectional association between serum 25(OH)D and the number of depressive symptom score, adjusted for age, sex, BMI, education, smoking, physical activity, alcohol intake, season of blood sampling, centre, and self-reported diabetes

Restricted cubic spline regression showed a modest dose–response association between serum 25(OH)D and the score of depressive symptoms, levelling off around 60–65 nmol/L (Fig. 2). Accordingly, testing for nonlinearity indicated that this association did not follow a linear tendency (*P* for nonlinearity: 0.04). Fully adjusted Poisson’s regression (model 2) subsequently showed a 22 % (RR 0.78, 95 % CI 0.68–0.89), 21 % (RR 0.79, 95 % CI 0.68–0.90), and 18 % (RR 0.82, 95 % CI 0.71–0.95) lower score of depressive symptoms in people in the second, third, and fourth quartiles of serum 25(OH)D, respectively, when compared to people in the first quartile (Table 2). Additionally including self-reported diabetes and stroke/TIA did not substantially change the results: RR 1.0 (ref) for the first quartile, RR 0.72 (95 % CI 0.62–0.84) for the second quartile, RR 0.74 (95 % CI 0.63–0.87) for the third quartile, and RR 0.84 (95 % CI 0.71–0.99) for the upper quartile, *P* for trend 0.0002. Prospective analyses using baseline serum 25(OH)D and depression data obtained after 2 years of vitamin D supplementation of 15 µg per day showed 26, 18, and 21 % less depressive symptoms in people in the second, third, and fourth quartiles of serum 25(OH)D, respectively, when compared to people in the first quartile (model 2; Table 2). Adjustment for treatment group did not substantially alter the results. Associations between vitamin D-related genetic make-up and the score of depressive symptoms were non-significant (supplementary Table I). Moreover, no significant interactions between serum 25(OH)D and any of the vitamin D-related genes were observed in association with the score of depressive symptoms.

**Table 2:** Associations between serum 25(OH)D at baseline with the number of depressive symptom score at baseline and after 2 years of vitamin D supplementation with 15 µg/day, relative risks (95 % CI)

	*Q*1	*Q*2	*Q*3	*Q*4	*P* for trend
25(OH)D status (nmol/L)	<36.7	36.7–53.3	53.4–71.7	>71.7	
Baseline, *n*	704	714	709	712	
Crude model (*n* = 2839)	1.0	0.69 (0.60–0.79) *P* < 0.0001	0.69 (0.61–0.79) *P* < 0.0001	0.68 (0.60–0.78) *P* < 0.0001	*P* < 0.0001
Model 1 (*n* = 2839)	1.0	0.76 (0.66–0.87) *P* < 0.0001	0.77 (0.67–0.88) *P* = 0.0001	0.78 (0.68–0.90) *P* = 0.0007	*P* < 0.0001
Model 2 (*n* = 2822)	1.0	0.78 (0.68–0.89) *P* = 0.0003	0.79 (0.68–0.90) *P* = 0.0007	0.82 (0.71–0.95) *P* = 0.01	*P* < 0.0001
Model 3 (*n* = 2183)	1.0	0.71 (0.61–0.83) *P* < 0.0001	0.73 (0.62–0.86) *P* < 0.0001	0.83 (0.70–0.98) *P* = 0.03	*P* = 0.0001
Follow-up, *n*	610	639	635	660	
Crude model (*n* = 2544)	1.0	0.66 (0.58–0.76) *P* < 0.0001	0.74 (0.64–0.85) *P* < 0.0001	0.68 (0.59–0.78) *P* < 0.0001	*P* < 0.0001
Model 1 (*n* = 2544)	1.0	0.72 (0.64–0.84) *P* < 0.0001	0.82 (0.71–0.95) *P* = 0.007	0.78 (0.67–0.90) *P* = 0.0005	*P* < 0.0001
Model 2 (*n* = 2531)^a^	1.0	0.74 (0.64–0.84) *P* < 0.0001	0.82 (0.70–0.95) *P* = 0.007	0.79 (0.68–0.92) *P* = 0.002	*P* < 0.0001
Model 3 (*n* = 1996)^a^	1.0	0.68 (0.59–0.79)	0.78 (0.66–0.93)	0.76 (0.64–0.90)	*P* < 0.0001

Model 1 is adjusted for age and sex

Model 2 is adjusted for age, sex, BMI, education, smoking, physical activity, alcohol intake, season of blood sampling, and centre

Model 3 is adjusted for age, sex, BMI, education, smoking, physical activity, alcohol intake, season of blood sampling, centre, and self-reported diabetes

^a^The associations at 2 years of follow-up were additionally adjusted for intervention group

### Depressive symptoms: is there an interplay between serum 25(OH)D and diabetes?

As vitamin D deficiency has been suggested to be a potential modifiable risk factor for diabetes [7], and diabetics have been shown to be at an increased risk of depression [8], also the potential mediation and modification effects of diabetes were examined. First of all, the association between serum 25(OH)D and self-reported diabetes was explored, showing strong associations over quartiles of serum 25(OH)D. Crude models showed an up to 42 % lower probability of having diabetes in the upper quartile, PR 0.58 (95 % CI 0.41–0.82). In the fully adjusted model, the associations attenuated, which was mainly attributable to the inclusion of BMI [PR 0.77 (95 % CI 0.52–1.14) in the upper quartile] (Table 3). The fully adjusted model that did not include BMI, which was investigated because BMI might be an intermediate in the association between 25(OH)D and diabetes, indicated a 43 % lower probability of having diabetes in the upper 25(OH)D quartile. Second, we studied the possible association between self-reported diabetes and the score of depressive symptoms, showing that participants with diabetes (*n* = 231) reported 17 % higher score of depressive symptoms than participants without diabetes (*n* = 2024) after full adjustment, RR 1.17 (95 % CI 1.00–1.38; Table 4). Mediation was further examined by extending the model of serum 25(OH)D and the score of depressive symptoms with self-reported diabetes, showing that the observed associations even slightly strengthened. Including the interaction term serum 25(OH)D*diabetes did not point towards a modification effect in association with the score of depressive symptoms (*P* for interaction = 0.82). Furthermore, removing BMI from the fully adjusted model did also not affect the association between 25(OH)D concentration and the score of depressive symptoms, specifically the RR with 95 % CI for model 2 without BMI was: *Q*1 = 1.0 (ref), *Q*2 = 0.77 (0.68–0.89) *P* = 0.0002, *Q*3 = 0.78 (0.68–0.90) *P* = 0.0004, and *Q*4 = 0.80 (0.69–0.93) *P* = 0.003. Including the interaction term of serum 25(OH)D*BMI did also not point towards a modification effect in association with the score of depressive symptoms (*P* for interaction = 0.11), nor did stratification of the cross-sectional associations by BMI indicate effect modification (Supplementary Table II).

**Table 3:** Associations between serum 25(OH)D and self-reported diabetes, PRs (95 % CI)

25(OH)D status (nmol/L)	*n*	*Q*1	*Q*2	*Q*3	*Q*4	*P* for trend
		4.1–36.7	36.7–53.4	53.4–71.7	71.7–193.6	
Diabetes, *n* (cases)		569 (79)	560 (55)	543 (54)	536 (43)	
Crude model	2208	1.0	0.71 (0.51–0.98)	0.72 (0.52–0.99)	0.58 (0.41–0.82)	0.003
Model 1	2208	1.0	0.68 (0.49–0.95)	0.69 (0.50–0.96)	0.56 (0.39–0.80)	0.002
Model 2	2191	1.0	0.80 (0.57–1.11)	0.81 (0.57–1.16)	0.77 (0.52–1.14)	0.20

Model 1 is adjusted for age and sex. Model 2 is adjusted for age, sex, BMI, education, smoking, physical activity, alcohol intake, season of blood sampling, and centre

**Table 4:** Associations of self-reported diabetes with the number of depressive symptom score at baseline, analysed with Poisson’s regression resulting in relative risks (95 % CI)

	Self-report diabetes	
	No (*n* = 2024)	Yes (*n* = 231)
Crude model	1.0	1.22 (1.04–1.43)
Model 1	1.0	1.25 (1.07–1.46)
Model 2	1.0	1.17 (1.00–1.38)

Model 1 is adjusted for age and sex. Model 2 is adjusted for age, sex, BMI, education, smoking, physical activity, alcohol intake, season of blood sampling, and centre

## Discussion

This study showed a clear cross-sectional association between serum 25(OH)D and the score of depressive symptoms as reported by the Geriatric Depression Scale-15. This association remained after 2 years of vitamin D supplementation. No significant associations between vitamin D-related genetic make-up and the score of depressive symptoms were observed, nor did we observe significant interactions between vitamin D-related genes and 25(OH)D concentrations. There was also no evidence for a potential mediation or modification effect by the presence of self-reported diabetes.

### A broader perspective

Several other observational studies also examined the association between 25(OH)D and depression in aged populations ≥60 years [9–15]. A study amongst 1282 Dutch men and women aged 65–95 years from the Longitudinal Aging Study Amsterdam observed that persons in the highest 25(OH)D quartile had a lower probability of depression as measured with the Center for Epidemiological Studies Depression Scale (CES-D) (*β* − 1.33 *P* = 0.03) when compared to the lowest quartile [12]. The Os study, including 883 Chinese men ≥65 years, also showed beneficial associations between 25(OH)D and depression, with an OR for depression of 0.46 (95 % CI 0.22–0.98, *P* for trend = 0.004) in the highest quartile after adjustment for age, BMI, education, physical activity, number of activities of daily living, diet quality index, smoking, alcohol consumption, season, number of chronic diseases, cognitive performance, and serum (ln)PTH concentration [11]. Another large study that observed a beneficial association between 25(OH)D and depression was the Health Survey for England, which used data of 2070 men and women ≥65 years [15]. To the best of our knowledge, none of the aforementioned studies accounted for gene profiles. We did, but we did not observe any association between vitamin D-related genetic make-up and the score of depressive symptoms, or interactions between 25(OH)D concentrations and vitamin D-related make-up. Kuningas and colleagues also explored associations between several VDR polymorphisms and observed an association between ApaI and depressive symptoms in 563 Dutch Caucasian older adults [26]. However, as we did not observe any association between the vitamin D genes, we do consider the possibility that it is not a higher 25(OH)D concentration that is responsible for a lower score of depressive symptoms, but that the observed association is explained by reverse causation or another factor that we could not control for in our analyses. Randomized controlled trials (RCTs) can provide more conclusive evidence on the direction of the association. However, to date, RCTs results are inconclusive. It has been argued that biological flaws may be the reason for the indecisive evidence, since several studies did not measure 25(OH)D concentrations, included participants with high 25(OH)D concentrations or used a relatively low dose of vitamin D [27].

### Underlying mechanisms

A low vitamin D concentration may predispose to depression through several biological mechanisms. Vitamin D has amongst others been linked to an increase in serotonin production [28] and a decrease in glucocorticoid-induced hippocampal cell death [29]. Vitamin D has furthermore been hypothesized to play a role in synthesis of neurotrophins, production of acetylcholine and glutathione, and down-regulation of L-type voltage-sensitive calcium channel expression (reviewed in [5]). Vitamin D may also indirectly fight depressive symptoms via its proposed anti-inflammatory effect [30]. To further explore the underlying mechanisms, we investigated the possible role of diabetes. Due to a variety of disease-related stress factors, diabetics are considered to be at increased risk of developing depression [8]. With that, low serum 25(OH)D concentrations may also predispose to glucose intolerance [7]. Thus, mediation as well as modification effects could be expected to be present. Nevertheless, fully adjusted models did not support an association between 25(OH)D and self-reported diabetes. In addition, adding self-reported diabetes to the model on 25(OH)D and depression did not attenuate the observed association. Interaction analyses furthermore indicated that there was no difference in the score of depressive symptoms when having diabetes or not. All in all, we therefore conclude that our data do not support a mediation or modification effect of diabetes in the vitamin D–depression pathway.

### Methodological considerations

A well-known limitation of observational studies is that it is not possible to say something about causality, specifically is the so-called exposure an actual risk factor or is it merely a consequence of the disease? Fortunately, we had the possibility to further explore this association in a longitudinal fashion, showing that 2 years of vitamin D supplementation with 15 µg per day did not change the association as observed at baseline. Interpreting these findings, however, is quite challenging, first of all because 25(OH)D concentrations were not measured after 2 years of follow-up. It may be suggested that vitamin D supplementation did not beneficially affect the score of depressive symptoms and that the association is explained by an unknown other factor that is strongly correlated with 25(OH)D status. On the other hand, it may also be that participants in the lowest quartile did reach higher 25(OH)D concentrations, but that these concentrations were still not high enough to decrease their probability of having a higher score of depressive symptoms relative to the other groups. Secondly, during 2 years of follow-up, participants may also have used vitamin D supplements on own initiative or medications that affect vitamin D metabolism, which may have affected the results. Another limitation of this study is that data were obtained from the B-PROOF study, an intervention trial examining the impact of vitamin B_12_ and folic acid on a variety of outcomes, which may have interfered with our longitudinal analyses. Adjusting the associations for treatment group, however, did not substantially alter the associations. This study is also limited by the fact diabetes diagnosis is based on self-report. Strengths of this study were the large study population, possibility to study associations after 2 years of vitamin D supplementation, opportunity to adjust for important confounders, as well as the possibility to investigate the role of diabetes and of vitamin D-related genes that have been linked to vitamin D synthesis, metabolism, and vitamin D receptor action.

## Conclusion

Our data support previously reported cross-sectional associations between higher 25(OH)D concentrations and a decreased risk of depression. Our subsequent finding that 2 years of vitamin D supplementation did not translate into a shift towards less depressive symptoms, however, raises concern about the temporality and causality of the association. No mediation or modification effect by diabetes was observed. To define the direction of the link between serum 25(OH)D and depression, there is a continuous need for prospective studies as well as well-designed RCTs.

## Electronic supplementary material

Below is the link to the electronic supplementary material.

Supplementary material 1 (DOCX 18 kb)
