# Supplementary material for: Low vitamin D status is associated with more depressive symptoms in Dutch older adults
Source: Eur J Nutr. 2015 Jul 4;55:1525–34. doi: 10.1007/s00394-015-0970-6 (PMC4875055; doi:10.1007/s00394-015-0970-6)
Supplement: Supplementary file 1 — Supplementary material 1 (DOCX 18 kb) [file 394_2015_970_MOESM1_ESM.docx]

**Supplementary Table I.** Associations between vitamin D related genetic make-up with the number of depressive symptom score at baseline and after 2-years of vitamin D supplementation with 15 µg/day, Relative Risks (95% CI)

|  | Continuous | Minor allele homozygotes | Heterozygotes | Major allele homozygotes |
| --- | --- | --- | --- | --- |
| Baseline (n=2555) |  |  |  |  |
| DHCR7 | 0.00±0.04 (P=0.99) | 1.0 | 0.96 (0.78-1.17) | 0.98 (0.80-1.19) |
| CYP2R1 | -0.02±0.04 (P=0.52) | 1.0 | 1.01 (0.87-1.18) | 0.96 (0.82-1.13) |
| CYP24A1 | 0.03±0.05 (P=0.58) | 1.0 | 0.93 (0.71-1.22) | 0.98 (0.75-1.28) |
| GC | -0.07±0.04 (P=0.11) | 1.0 | 0.93 (0.77-1.14) | 0.87 (0.72-1.06) |
| TaqI / BsmI | 0.01±0.04 (P=0.75) | 1.0 | 1.03 (0.88-1.21) | 1.03 (0.87-1.22) |
| ApaI | -0.01±0.04 (P=0.79) | 1.0 | 1.03 (0.91-1.17) | 0.98 (0.85-1.14) |
| Cdx2 | 0.01±0.05 (P=0.79) | 1.0 | 1.01 (0.79-1.32) | 1.02 (0.79-1.32) |
| Follow-up (n=2307) |  |  |  |  |
| DHCR7 | 0.04±0.05 (P=0.35) | 1.0 | 1.02 (0.81-1.28) | 1.07 (0.86-1.35) |
| CYP2R1 | -0.02±0.04 (P=0.68) | 1.0 | 1.03 (0.88-1.20) | 0.98 (0.83-1.15) |
| CYP24A1 | -0.04±0.05 (P=0.46) | 1.0 | 0.96 (0.71-1.29) | 0.92 (0.69-1.24) |
| GC | -0.04±0.04 (P=0.36) | 1.0 | 0.83 (0.69-1.01) | 0.85 (0.71-1.03) |
| TaqI / BsmI | 0.03±0.04 (P=0.51) | 1.0 | 1.02 (0.87-1.20) | 1.06 (0.89-1.25) |
| ApaI | -0.04±0.04 (P=0.27) | 1.0 | 0.97 (0.85-1.12) | 0.92 (0.78-1.08) |
| Cdx2 | -0.02±0.05 (P=0.75) | 1.0 | 0.87 (0.65-1.15) | 0.88 (0.67-1.17) |

**Supplementary Table II.** Associations between serum 25(OH)D at baseline with the number of depressive symptom score at baseline stratified for BMI, Relative Risks (95% CI)

|  | *BMI<25 kg/m^2^* | | | | | *BMI≥25 kg/m^2^* | | | | |
| --- | --- | --- | --- | --- | --- | --- | --- | --- | --- | --- |
|  | Q1 | Q2 | Q3 | Q4 | *P for trend* | Q1 | Q2 | Q3 | Q4 | *P for trend* |
| ***25(OH)D (nmol/L)*** | <36.7 | 36.7-53.3 | 53.4-71.7 | >71.7 |  | <36.7 | 36.7-53.3 | 53.4-71.7 | >71.7 |  |
| n | 199 | 198 | 179 | 252 |  | 501 | 210 | 526 | 459 |  |
| Crude model (n=828) / (1996) | 1.0 | 0.64 (0.50-0.83)  P=0.0006 | 0.66 (0.52-0.84)  P=0.0007 | 0.64 (0.51-0.81)  P=0.0002 | P<0.0001 | 1.0 | 0.71 (0.60-0.83)  P<0.0001 | 0.70 (0.59-0.82)  P<0.0001 | 0.70 (0.59-0.83)  P<0.0001 | P<0.0001 |
| Model 1 (n=828) / (1996) | 1.0 | 0.75 (0.57-0.98)  P=0.04 | 0.77 (0.60-1.00) P=0.05 | 0.77 (0.60-1.00)  P=0.05 | P=0.002 | 1.0 | 0.76 (0.65-0.89)  P=0.0008 | 0.76 (0.64-0.89) P=0.0008 | 0.79 (0.66-0.93)  P=0.007 | P<0.0001 |
| Model 2 (n=828) / (n=1994) | 1.0 | 0.78 (0.60-1.00) P=0.05 | 0.82 (0.65-1.05) P=0.12 | 0.81 (0.64-1.04) P=0.10 | P=0.02 | 1.0 | 0.78 (0.66-0.91) P=0.002 | 0.77 (0.65-0.91) P=0.002 | 0.79 (0.66-0.95) P=0.01 | P<0.0001 |

Model 1 is adjusted for age and sex. Model 2 is adjusted for age, sex, education, smoking, physical activity, alcohol intake, season of blood sampling, and centre.

*European Journal of Nutrition*

**Vitamin D-epressed: Low vitamin D status is associated with more depressive symptoms in Dutch older adults.**

EM Brouwer-Brolsma PhD, RAM Dhonukshe-Rutten PhD, JP van Wijngaarden PhD, NL van de Zwaluw PhD, E. Sohl MSc, PH in ’t Veld MSc, SC van Dijk MD, KMA Swart MSc, AW Enneman PhD, AC Ham MSc, NM van Schoor, PhD, N van der Velde PhD, AG Uitterlinden PhD, P Lips PhD, EJM Feskens PhD, LCPGM de Groot PhD
